# The role of dieting, happiness with appearance, self-esteem, and bullying in the relationship between mental health and body-mass index among UK adolescents: a longitudinal analysis of the Millennium Cohort Study

**DOI:** 10.1016/j.eclinm.2023.101992

**Published:** 2023-05-22

**Authors:** Hanna Creese, Sonia Saxena, Dasha Nicholls, Ana Pascual Sanchez, Dougal Hargreaves

**Affiliations:** a School of Public Health, Imperial College London, London, UK; b Department of Brain Sciences, Imperial College London, UK

**Keywords:** Dieting, Happiness with appearance, Body image, Self-esteem, Bullying, Mental health, Body-mass index

## Abstract

**Background:**

Mental illness and obesity are among the biggest challenges to population health, they are linked, and may be modifiable during adolescence. We aimed to determine intervening pathways between mental health and BMI z-score symptoms across adolescence.

**Methods:**

In this longitudinal cohort study, we used path models to examine self-reported dieting, happiness with appearance, self-esteem and bullying at 14 years as potential mediators of the cross-lagged relationship between mental health (via the Strengths and Difficulties Questionnaire) and Body Mass Index (BMI) z-score at 11 and 17 years by sex in the UK Millennium Cohort Study, a prospective cohort study of 18,818 children born in the UK between September 1st, 2000, and January 31st, 2002. Full, incomplete data on all singleton children still participating in the study by age 11 years were analysed in GSEM via maximum likelihood estimation (N = 12,450).

**Findings:**

We found happiness with appearance and self-esteem, but not dieting or bullying, mediated the relationship between BMI age 11 and mental health age 17. Each increase in BMI z-score at 11 years was associated with 0.12 increase for boys and a 0.19 increase for girls in scores of unhappiness with appearance (boys: *b* 0.12, 95% C.I.; girls *b* 0.19, C.I. 0.14 to 0.23) and a 16% increase for boys and a 22% increase for girls in odds of low self-esteem (boys OR 1.16, 95% C.I. 1.07 to 1.26; girls: OR 1.22, 95% C.I. 1.15 to 1.30) at 14 years. In turn, for both boys and girls, being unhappy with appearance and low self-esteem at 14 years were associated with a greater likelihood of emotional and externalizing symptoms at 17 years.

**Interpretation:**

Early prevention strategies to encourage healthy physical and mental development of children need to focus on the promotion of positive body-mage and self-esteem.

**Funding:**

The 10.13039/501100000272National Institute for Health and Care Research (10.13039/501100000272NIHR) 10.13039/501100012349School for Public Health Research (10.13039/501100012349SPHR).

**Research in context**

**Evidence before this study**

We systematically searched MEDLINE for studies about mental health and Body Mass Index (BMI) in adolescents published in English between database inception and August 1st, 2022, using the following search terms: “mental health”, “overweight”, “obesity”, “BMI” “internalising”, “externalizing”, “dieting”, “self-esteem”, “body satisfaction”, “body image”, and “bullying”. Studies suggested a cyclical relationship between mental health and BMI during adolescence which varied somewhat by sex. Several studies identified plausible potential explanations for links between mental health and BMI, including effects on dieting, body satisfaction, self-esteem, and experiences of bullying. To our knowledge, no prior studies have examined all these potential explanations simultaneously whilst accounting for longitudinal directional effects in the relationship between mental health symptoms and BMI by sex.

**Added value of this study**

Using large scale, longitudinal data that is generalisable to the wider UK population on more than 12,000 adolescents, we examined multiple pathways simultaneously between 11 and 17 years finding happiness with appearance and self-esteem to be intervening pathways between BMI z-score and mental health symptoms. For boys and girls, each increase in BMI z-score predicted being less happy with appearance and greater odds of low self-esteem later in adolescence. In turn, unhappiness with appearance and low self-esteem was associated later with greater odds of emotional and externalizing symptoms for both boys and girls, and higher BMI for boys. Having emotional symptoms was associated with later increased odds of low self-esteem and unhappiness with appearance for boys and girls and bullying for girls. For boys and girls, experiencing externalizing symptoms was associated with greater odds of frequent bullying and frequent bullying was associated with greater odds of emotional and externalizing symptoms later in adolescence. For boys, experiencing externalizing symptoms was also associated with low self-esteem.

**Implications of all the available evidence**

Happiness with appearance and self-esteem are important pathways via which BMI is associated with mental health symptoms in young people. Findings are highly relevant for the national curriculum promoting healthy body image and self-esteem, calls on industry and social media to endorse healthy body image, and policies to reduce weight stigma.

## Introduction

Adolescence (10–19 years) is a formative time in which it is crucial to promote mental and physical health to protect against lifetime risks of mental illness and obesity.^1^ As children get older, evidence supports an interrelationship between high Body Mass Index (BMI, kg/m^2^) and mental health difficulties, with earlier obesity associated with later emotional symptoms, including anxiety and depression, and earlier emotional symptoms associated with later obesity^2^, ^3^, ^4^ into adulthood.^5^ There is also robust cross-sectional evidence for the association between overweight/obesity and externalizing behaviours, including aggression and impulsivity.^6^^,^^7^ However, the evidence of a longitudinal association is less reliable and conclusions on the direction of the association cannot be drawn. Addressing problems of obesity and poor mental health are among the biggest challenges to public health globally.^8^^,^^9^ While early intervention efforts may benefit from targeting both health outcomes, the mechanisms for this bidirectional relationship are incompletely understood. A better understanding of the psychosocial factors contributing to both poor mental health and overweight/obesity in adolescence has the potential to meaningfully contribute to prevention strategies for both and promote lifelong health.

There are psychosocial mechanisms which might help explain the relationship between being obese and having poor mental health and potential causality in both directions. There is strong evidence that by adolescence, there is an increased risk of low self-esteem and body image concerns in young people with obesity.^10^ Weight stigma increases vulnerability to low self-esteem^11^ and poor body image.^12^^,^^13^ In turn, low self-esteem and body dissatisfaction can lead to depressive symptoms in adolescents.^14^, ^15^, ^16^ Negative body image is harmful from the perspective of weight management as well as mental health^17^ with dieting adolescents often gaining more weight than those not dieting.^18^ However, to our knowledge, prior research has not examined these potential explanatory pathways in the cyclical relationship between mental health and obesity whilst accounting for longitudinal directional effects.

Body weight is the most common reason that young people are bullied.^11^^,^^19^ The experience of bullying has long-term impacts on emotional and externalizing symptoms into adulthood.^20^^,^^21^ There is also evidence that having mental health difficulties, in particular externalizing difficulties,^22^ increases the risk of being bullied in adolescence. Therefore, bullying may also mediate the cyclical relationship between mental health and obesity. Cross-sectional analysis has shown that bullying may be an important factor in the relationship between weight status and mental health.^23^ Yet, longitudinal analysis unpicking the direction of pathways is needed.

Girls appear to be more at risk from obesity and mental health difficulties than boys.^24^ It has been suggested that girls face more stigma for excess weight than boys,^25^ linked to sociocultural pressures of thinness and beauty ideals. In addition, women are more likely to engage in ruminative coping which is linked to depression.^24^ Together, these factors may lead girls at a higher weight to be more vulnerable to mental health difficulties, compared to boys.

**Fig. 1:**
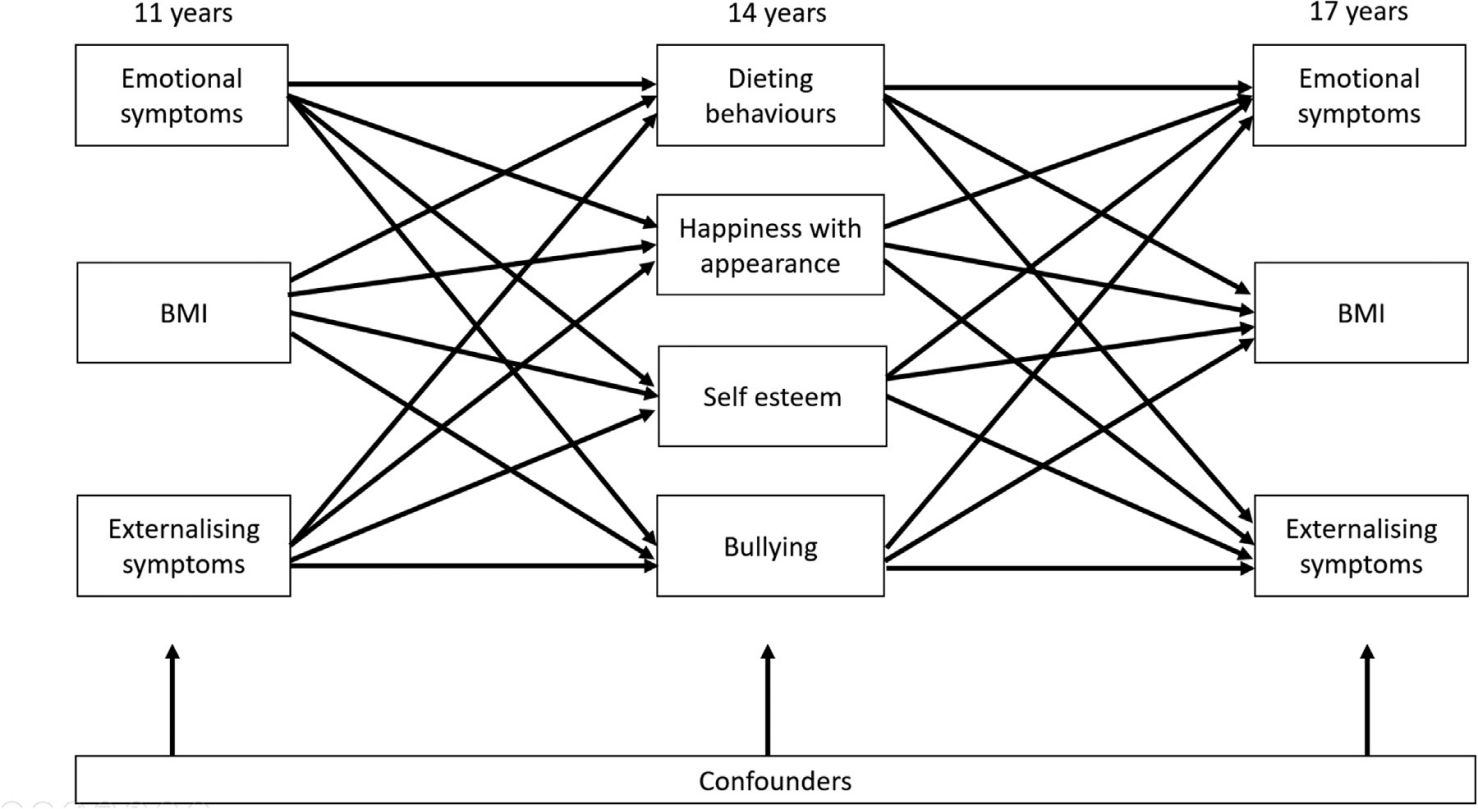
**Logic model of mediating paths at 14 years (dieting behaviours; happiness with appearance; self-esteem; bullying) of cross-lagged GSEM between BMI Z-score, emotional and externalizing symptoms between age 11 and 17 years stratified by sex**. Notes: Models adjust for cross–sectional associations between BMI Z-score, emotional and externalizing symptoms, and confounders: household income; maternal education; child ethnicity, early puberty, and family structure.

To improve understanding of the mechanisms at play, in this paper we use the UK representative Millennium Cohort Study (MCS) to simultaneously test dieting, happiness with appearance, self-esteem, and bullying as potential mediators of the cross-lagged relationship between BMI and mental health across adolescence by sex (Fig. 1). For both, boys and girls, we hypothesize that greater BMI at 11 years will be associated with later mental health difficulties by 17 years, and vice versa, via increases in dieting behaviours, unhappiness with appearance, low self-esteem, and frequent bullying.

## Methods

### Study design and population

The Millennium Cohort Study (MCS) is a prospective cohort study of 18,818 children born in the UK between September 2000 and January 2002.^26^ At every data collection sweep written informed consent was obtained from parental respondents, and from the age 11 sweep onwards, from cohort members themselves. The MCS was approved by the South West and London Multi-Centre Research Ethics Committees, has been fully anonymised and did not require additional ethics review for our study.^27^ Participating families were randomly sampled from all four countries of the UK, with a stratified cluster sampling design to ensure the representation of disadvantaged and ethnically diverse areas. The first data collection sweep was when cohort members were approximately 9 months of age and the subsequent six sweeps of data were collected at ages 3, 5, 7, 11, 14 and 17 years. We included all singleton children in the study between 11 and 17 years. We have adhered to the Strengthening the Reporting of Observational Studies in Epidemiology (STROBE) reporting guidelines for cohort studies.

### Measures

#### Emotional symptoms

Emotional symptoms for each child were defined using the emotional problems subscale of the widely validated, by sex and at multiple ages,^28^^,^^29^ and extensively used, Strengths and Difficulties Questionnaire (SDQ) reported by a parent or guardian (>95% were mothers)^30^ captured at 11 and 17 years. The scale consists of 5 items (e.g., often seems worried; often seems unhappy, downhearted, tearful), rated as not true 0, somewhat true 1, or, certainly true 2, which were summed to create a total score ranging from 0 to 10. Cronbach's alpha indicated the items had acceptable internal consistency (Cronbach's alpha for boys 0.71 and for girls 0.71). As the distribution was non-normal, a binary variable was created following a recommended cut point for high symptoms indicating clinical significance (low/moderate 0–4 vs high symptoms 5–10).^31^

#### Externalizing symptoms

Externalizing symptoms were measured using the conduct problems (e.g., often loses temper; often fights with or bullies other children) and hyperactivity/inattention (e.g., easily distracted, concentration wanders; is restless, overactive, cannot stay still) subscales SDQ reported by a parent or guardian at 11 and 17 years. Both scales consist of 5 items, rated as not true 0, somewhat true 1, or, certainly true 2, which are summed together to create a total score ranging from 0 to 20. Cronbach's alpha indicated the items had acceptable internal consistency (Cronbach's alpha for boys 0.82 and for girls 0.78). As the distribution was non-normal, a binary variable was created following recommended a cut point for higher symptoms indicating clinical significance (low/moderate 0–10 vs high symptoms 11–20).^31^

#### BMI Z-score

To calculate Body Mass Index (BMI, kg/m^2^), children's heights and weights without shoes and outdoor clothing using Tanita™ scales were measured during home visits by trained interviewers at 11 and 17 years. Weights in kilograms to 1 decimal place and heights to the nearest millimetre were recorded. BMI was then standardised by sex and age using World Health Organization (WHO) international reference data via the Zanthro command in Stata.^32^ In descriptive analyses, sex and age-specific cut points as defined by the International Obesity Taskforce (IOTF)^33^ categorized children's BMI as not-overweight/obese, overweight or obese.

### Potential mediators

#### Dieting behaviours

Dieting behaviour was assessed using two items at age 14 years: “Have you ever exercised to lose weight or to avoid gaining weight?” (0 no and 1 yes) and “Have you ever eaten less food, fewer calories, or foods low in fat to lose weight or to avoid gaining weight?” (0 no and 1 yes). Answers were combined to create three categories: 0 no dieting behaviours, 1 dieting behaviour, and 2 both dieting behaviours.

#### Happiness with appearance

Happiness with appearance was assessed by an item which asked children: “On a scale of 1–7 where 1 means completely happy and 7 means not at all happy, how do you feel about the way you look?” (taken from Chan and Koo's (2010) to scale measure happiness^34^).

#### Self-esteem

Self-esteem was assessed using the items from the Rosenberg self-esteem scale at age 14 years^35^: I am satisfied with myself; have good qualities; able to do things similar to others; a person of value; and feel good about oneself. Each item was rated as: strongly agree 1, agree 2, disagree 3, or strongly disagree 4. The sum of these items resulted in a score between 1 and 20 for each child, with higher scores indicative of low self-esteem. Cronbach's alpha indicated the items had acceptable internal consistency (Cronbach's alpha 0.90). The Rosenberg scale showed scale had a distinctly non-normal distribution for which no transformation was satisfactory. Therefore, a dichotomised variable (high/moderate vs low) was derived with a cut point of 11 (i.e., the top 20% of the distribution) to indicate low self-esteem.

#### Bullying

Bullying was assessed using two items at age 14 years: “How often have other children sent you unwanted or nasty emails, texts or messages or posted something nasty about you on a website?”, and “How often do other children hurt you or pick on you on purpose?”, with responses ranging from most days; about once a week; about once a month; every few months; less often; never. Combined responses were reverse coded, and the total sum resulted in a score between 1 and 12 for each child, with higher scores indicative of more frequent bullying. Treating bullying as continuous was not tenable due to non-normal distribution. Therefore, a dichotomised variable (low/moderate vs high frequency) was derived with a cut point of 5 (i.e., the top 20% of the distribution) to indicate high levels of bullying.

### Potential confounders

We controlled for the following confounders captured at 11 years in our analysis: household income represented in Organisation for Economic Co-operation and Development UK equivalized quintiles; highest educational qualification attained by mother 1) higher degree or first-degree qualifications, 2) diploma in higher education, 3) A levels (exams usually taken around 18 years), 4) General Certificate of Secondary Education (GCSE, exams that are usually taken around age 16 years) grades A–C, 5) GCSE grades D–G, or 6) None of these qualifications; child ethnicity and early puberty status (whether menstruation has begun for girls; whether facial hair or voice deepening has begun for boys), and family structure (two vs one parent).

### Statistical analysis

First, we examined the proportion of children with high emotional or externalizing symptoms at 11 years by sample characteristics using chi-squared tests. Next, we examined mean BMI z-score at 11 years by other sample characteristics using t-tests and one-way ANOVA tests. Then used cross-lagged Generalized Structural Equation Models (GSEM) to estimate longitudinal pathways from BMI, emotional, and externalizing symptoms at 11 years to BMI, emotional, and externalizing symptoms at 17 years, while accounting for cross–sectional associations at baseline (Fig. 1). Tests of sex interaction for emotional and externalizing symptoms with BMI were significant (*p* value < 0.001). Therefore, all analyses were stratified by sex. MCS sampling and response weights were used to account for sampling design and attrition up to the 11-year survey. Full, incomplete data were analysed in GSEM via maximum likelihood estimation. We adjusted all models for potential confounders. All analyses were conducted using Stata 16.1.

### Role of the funding source

The funder of the study had no role in study design, data collection, data analysis, data interpretation, or writing of the report. HC and DH had access to the data and final responsibility for the decision to submit for publication.

## Results

### Participants

There were 18,296 singleton children included in the first MCS data collection sweep at age 9 months. By age 11 years 13,112 singleton children were still participating in the study. Of these children, 12,450 (95%) had some data on the variables so were included in GSEM analyses. The sample were evenly distributed between boys and girls (boys 51.7%; girls 48.3%) and majority white British ethnicity (Table 1).

**Table 2:** Mean BMI z-score at 11 and 17 years by characteristics of the study population.

	Mean BMI z-score at 11 years		Mean BMI z-score at 17 years	
	Boys	Girls	Boys	Girls
**Overall**	0.58	0.44	0.52	0.58
*P* value^a^		<0.001		0.0027
**Emotional symptoms at 11 years**				
Low/moderate	0.57	0.43	0.48	0.57
High	0.65	0.54	0.88	0.66
*P* value	0.015	0.026	0.0078	0.15
**Externalizing symptoms at 11 years**				
Low/moderate	0.59	0.44	0.48	0.57
High	0.50	0.53	0.96	0.79
*P* value	0.69	0.076	0.021	0.12
**Emotional symptoms at 17 years**				
Low/moderate	0.54	0.40	0.46	0.59
High	0.76	0.39	0.74	0.59
*P* value	0.085	0.57	0.29	0.13
**Externalizing symptoms at 17 years**				
Low/moderate	0.60	0.39	0.52	0.58
High	0.48	0.42	0.53	0.78
*P* value	0.06	0.53	0.42	0.027
**Child ethnicity**				
White	0.56	0.44	0.50	0.58
Mixed	0.71	0.46	0.43	0.84
South Asian	0.61	0.36	0.62	0.43
Black	0.84	0.88	0.96	1.07
*P* value	<0.013	<0.001	0.58	<0.001
**Early puberty**				
Not begun	0.53	0.36	0.49	0.54
Has begun	0.78	1.15	0.71	1.10
*P* value	<0.001	<0.001	<0.001	<0.001
**Household income quintile**				
Top	0.42	0.25	0.38	0.40
Fourth	0.56	0.40	0.61	0.62
Third	0.63	0.54	0.49	0.59
Second	0.67	0.58	0.48	0.69
Bottom	0.61	0.44	0.77	0.77
*P* value	<0.001	<0.001	0.012	<0.001
**Maternal education**				
Degree plus	0.43	0.23	0.35	0.42
Diploma	0.48	0.34	0.44	0.47
A levels	0.57	0.43	0.51	0.70
GCSE A-C	0.67	0.51	0.62	0.71
GCSE D-G	0.61	0.60	0.52	0.71
None	0.68	0.59	0.90	0.56
*P* value	<0.001	<0.001	0.0080	<0.001
**Family structure**				
Two parents	0.55	0.39	0.50	0.54
One parent	0.65	0.58	0.61	0.72
*P* value	<0.001	<0.001	0.18	<0.001
**Dieting behaviours**				
None	0.01	−0.30	−0.06	−0.02
One	0.66	0.40	0.65	0.59
Both	1.28	0.88	1.21	0.92
*P* value	<0.001	<0.001	<0.001	<0.001
**Happiness with appearance**				
1 Completely happy	0.45	0.27	0.40	0.48
2	0.49	0.15	0.30	0.33
3	0.62	0.34	0.53	0.52
4	0.78	0.52	0.81	0.62
5	0.57	0.52	0.54	0.75
6	0.91	0.68	1.13	0.70
7 Not at all happy	0.98	0.92	1.21	0.97
*P* value	<0.001	<0.001	<0.001	<0.001
**Self-esteem**				
High/moderate	0.54	0.31	0.46	0.47
Low	0.79	0.65	0.73	0.77
*P* value	<0.001	<0.001	0.0053	<0.001
**Bullying**				
Not bullied	0.58	0.42	0.51	0.57
Bullied	0.56	0.48	0.46	0.58
*P* value	0.58	0.15	0.98	0.15

Notes: Estimates are weighted with sample weights.

a *P* values for t-tests or one-way analysis of variance (ANOVA) comparing statistical differences between means.

### Prevalence of dieting, happiness with appearance, self-esteem, and bullying by BMI z-score and mental health symptoms

Overall, mean BMI z-score was 0.58 for boys and 0.44 for girls at 11 years and 0.52 for boys and 0.58 for girls at 17 years (Table 2). Both, boys and girls who had dieted (for example, boys at 11 years: no dieting *m* 0.01 vs both dieting behaviours *m* 1.28, *p* value < 0.001; girls at 11 years who had dieted *m* −0.30 vs both dieting behaviours *m* 0.88, *p* value < 0.001), were unhappy with their appearance (boys at 11 years who were completely happy with their appearance *m* 0.45 vs not at all happy *m* 0.98, *p* value < 0.001; girls who were happy *m* 0.27 vs not happy at all *m* 0.92, *p* value < 0.001), or had low self-esteem (boys: high/moderate *m* 0.54 vs low *m* 0.79, *p* value < 0.001; girls high/moderate *m* 0.31 vs low *m* 0.65, *p* value < 0.001) had higher mean BMI z-scores at 11 and 17 years (Table 2). There were no differences in BMI by bullying.

**Table 1:** Clinically relevant emotional and externalizing symptoms (percent) at 11 years by sample characteristics.

	Overall (n)		Emotional symptoms % (n)		Externalizing symptoms % (n)	
	Boys	Girls	Boys	Girls	Boys	Girls
**Overall**	51.7 (6634)	48.3 (6478)	11.7 (684)	12.2 (721)	11.7 (629)	5.4 (297)
*P* value				0.49		<0.001
**Weight status at 11 years**						
Healthy weight	38.4 (4387)	32.8 (3956)	10.3 (392)	10.8 (401)	11.5 (386)	5.0 (167)
Overweight	10.6 (1236)	11.6 (1389)	12.0 (136)	13.1 (165)	9.7 (102)	5.8 (76)
Obese	3.3 (395)	3.3 (413)	18.9 (68)	18.7 (68)	15.3 (56)	7.9 (24)
*P* value		<0.001	<0.001	<0.001	0.082	0.13
**Weight status at 17 years**						
Healthy weight	35.1 (2487)	33.8 (2478)	8.0 (197)	9.4 (232)	7.5 (159)	4.7 (88)
Overweight	9.6 (731)	10.4 (797)	12.2 (76)	11.2 (88)	8.1 (56)	3.9 (32)
Obese	5.7 (386)	5.5 (430)	21.1 (52)	14.4 (55)	26.1 (55)	7.5 (21)
*P* value		0.44	0.014	0.050	<0.001	0.33
**Child ethnicity**						
White	44.1 (5489)	41.6 (5348)	11.8 (575)	12.1 (608)	12.0 (540)	5.4 (251)
Mixed	1.9 (183)	1.6 (195)	12.6 (21)	20.5 (32)	12.5 (22)	10.2 (15)
South Asian	3.7 (638)	3.5 (643)	10.5 (60)	10.7 (59)	7.9 (45)	4.5 (27)
Black	2.0 (223)	1.5 (200)	8.8 (17)	8.4 (14)	8.8 (12)	2.1 (3)
*P* value for trend		0.24	0.55	0.018	0.30	0.031
**Early puberty**						
Not begun	43.4 (5428)	41.9 (5463)	11.5 (533)	11.8 (618)	11.6 (498)	5.0 (245)
Has begun	9.8 (1206)	5.0 (626)	12.4 (151)	15.4 (83)	11.8 (131)	9.0 (46)
*P* value		<0.001	0.47	0.042	0.90	<0.001
**Household income**						
Top	10.3 (1281)	9.8 (1254)	6.5 (76)	6.2 (75)	4.3 (46)	0.9 (7)
Fourth	10.4 (1411)	9.5 (1356)	9.0 (118)	10.5 (124)	6.3 (70)	3.2 (29)
Third	10.5 (1443)	9.5 (1350)	12.5 (148)	11.9 (147)	10.5 (133)	4.9 (52)
Second	10.3 (1269)	9.8 (1250)	13.2 (150)	15.0 (187)	12.9 (143)	8.6 (91)
Bottom	10.3 (1230)	9.7 (1268)	17.5 (192)	17.9 (188)	25.4 (237)	9.6 (118)
*P* value for trend		0.85	<0.001	<0.001	<0.001	<0.001
**Family structure**						
Two parents	37.8 (5047)	35.6 (4980)	10.9 (467)	10.5 (485)	9.5 (400)	4.4 (186)
One parent	13.9 (1587)	12.7 (1498)	13.9 (217)	17.1 (236)	17.5 (229)	8.1 (111)
*P* value		0.51	0.0079	<0.001	<0.001	<0.001
**Dieting behaviours**						
None	20.9 (2134)	13.5 (1491)	10.2 (191)	13.0 (159)	11.1 (163)	4.8 (3756
One	15.5 (1534)	11.4 (1220)	10.5 (144)	12.8 (124)	9.1 (115)	5.3 (41)
Both	14.9 (1517)	23.8 (2599)	12.0 (152)	12.4 (295)	12.2 (137)	5.0 (117)
*P* value		<0.001	0.44	0.93	0.17	0.94
**Happiness with appearance**						
1 Completely happy	9.2 (968)	4.1 (449)	11.9 (104)	9.9 (42)	11.4 (86)	6.8 (22)
2	15.9 (1628)	8.5 (977)	7.5 (108)	8.1 (75)	9.6 (104)	4.0 (30)
3	12.6 (1243)	10.8 (1185)	11.7 (123)	11.8 (108)	11.0 (96)	3.0 (29)
4	7.3 (736)	10.7 (1152)	11.4 (76)	12.6 (130)	10.8 (66)	4.4 (42)
5	3.8 (357)	6.9 (742)	14.1 (41)	14.2 (91)	11.3 (33)	5.3 (35)
6	1.6 (165)	4.9 (493)	15.4 (19)	19.6 (79)	10.7 (13)	8.9 (33)
7 Not at all happy	0.7 (67)	3.0 (302)	22.6 (11)	17.1 (50)	27.5 (11)	7.4 (21)
*P* value		<0.001	0.0031	<0.001	0.088	0.0050
**Self-esteem**						
High/moderate	42.7 (4363)	30.6 (3425)	9.6 (370)	10.3 (303)	9.9 (316)	4.0 (117)
Low	8.5 (812)	18.2 (1875)	17.2 (115)	16.5 (271)	15.0 (97)	6.7 (95)
*P* value		<0.001	<0.001	<0.001	0.0013	0.0035
**Bullying**						
Not bullied	41.0 (4195)	38.2 (4196)	10.1 (372)	11.2 (407)	9.8 (303)	3.6 (129)
Bullied	10.2 (1000)	10.6 (1114)	13.7 (116)	17.8 (171)	15.2 (113)	9.9 (84)
*P* value		0.092	0.019	<0.001	<0.001	<0.001

Notes: Estimates are weighted with sample weights. Sample sizes are unweighted. *P* values for chi-squared tests comparing the distribution of categorical variables.

Girls had a greater prevalence of dieting behaviours (both behaviours: girls 23.8%; boys 14.9%, *p* value < 0.001), unhappiness with appearance (not at all happy: girls 3.0%; boys 0.7%, *p* value < 0.001) and low self-esteem (girls 18.2%; boys 8.5%, *p* value < 0.001) than boys (Table 1). There were no sex differences for bullying (*p* value 0.092). There was no association between dieting and either emotional or externalizing symptoms for boys or girls (Table 1). Both, boys and girls who were unhappy with their appearance, had low self-esteem or experienced frequent bullying at 11 years, had a greater prevalence of emotional and externalizing symptoms.

### Prevalence of overweight and obesity by mental health symptoms

Boys and girls had a similar prevalence of emotional symptoms (boys 11.7%; girls 12.2%, *p* value 0.4942), but boys had significantly more externalizing symptoms than girls (boys 11.7%; girls 5.4%, *p* value < 0.001) at 11 years. Around 1 in 5 adolescents were overweight (boys 10.6%; girls 11.6%) and 6% had obesity (boys 3.3%; girls 3.3%) (Table 1). Both, boys and girls with obesity, had a greater prevalence of emotional symptoms at 11 years (boys with obesity 18.9% vs boys with healthy weight 10.3%; girls with obesity 18.7% vs girls with healthy weight 10.8%). Boys had double the prevalence of externalizing symptoms compared to girls at 11 years (boys 11.7% vs 5.4%). Boys with obesity had a greater prevalence of externalizing symptoms at 11 years (boys with obesity 15.3% vs boys with healthy weight 11.5%). Girl's weight status and externalizing symptoms were not associated (11 years *p* value 0.13).

Is there a cross-lagged relationship between mental health and BMI z-score across adolescence?

**Fig. 2:**
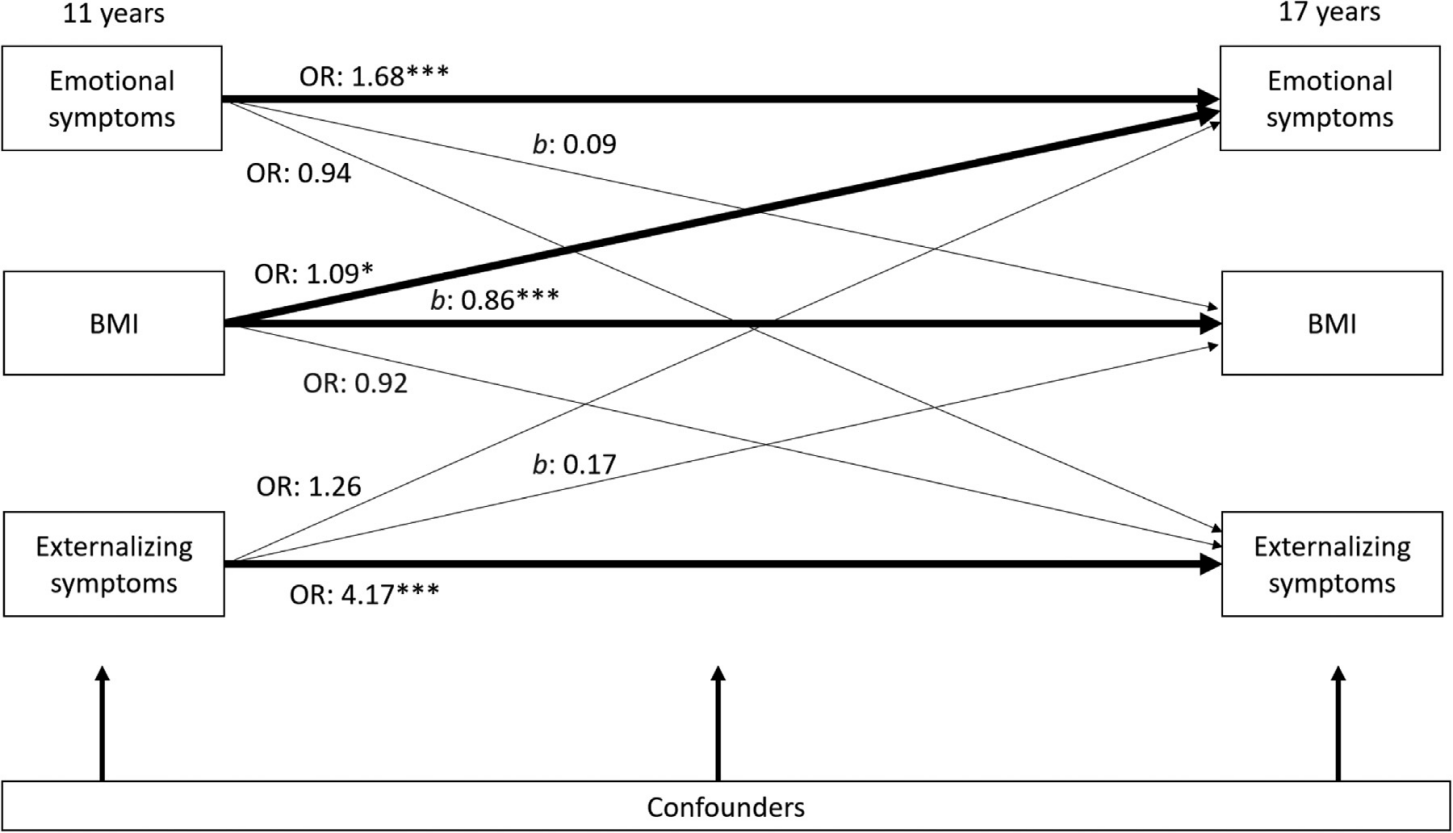
**Cross-lagged associations from 11 years to 17 years between BMI Z-score and mental health symptoms for boys (N** = **6417)**. Notes: Odds ratios [OR] for logistic regression; Coefficients [*b*] for linear regression. All regressions are stratified by sex and adjust for cross–sectional associations between BMI Z-score, emotional and externalizing symptoms at baseline, and potential confounders: household income; maternal education; child ethnicity, early puberty and family structure). Regression estimates are weighted with sampling and attrition weights. ∗*p* < 0.05, ∗∗*p* < 0.01, ∗∗∗*p* < 0.001.

For boys, we did not find evidence of a cross-lagged relationship between BMI z-score and mental health between 11 and 17 years. However, after adjustment for cross–sectional associations and potential confounders, each increase in BMI z-score at 11 years predicted a 9% higher odds ratio for emotional symptoms at 17 years (OR 1.09, 95% C.I. 1.01 to 1.18). BMI did not predict later externalizing symptoms (OR 0.92, 95% C.I. 0.82 to 1.03). Having externalizing or emotional symptoms at 11 years was not associated with later BMI at 17 years in boys (Fig. 2).

**Fig. 3:**
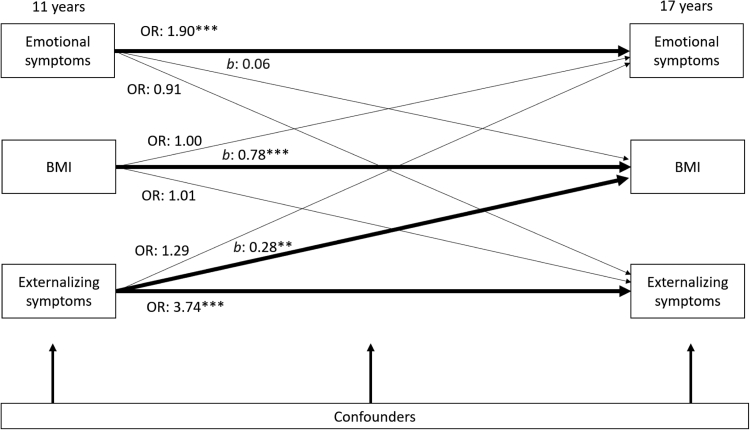
**Cross-lagged associations from 11 years to 17 years between BMI Z-score and mental health symptoms for girls (N** = **6091)**. Notes: Odds ratios [OR] for logistic regression; Coefficients [*b*] for linear regression. All regressions are stratified by sex and adjust for cross–sectional associations between BMI Z-score, emotional and externalizing symptoms at baseline, and potential confounders: household income; maternal education; child ethnicity, early puberty and family structure). Regression estimates are weighted with sampling and attrition weights. ∗*p* < 0.05, ∗∗*p* < 0.01, ∗∗∗*p* < 0.001.

For girls, after adjustment for cross–sectional associations and potential confounders, having externalizing symptoms at 11 years predicted a 0.28 increase in BMI z-score at 17 years (*b* 0.28, 95% C.I. 0.09 to 0.47). Emotional symptoms and BMI had no longitudinal association in either direction (Fig. 3).

Are dieting behaviours, happiness with appearance, self-esteem, and bullying potential mediators of the relationship between BMI z-score and mental health symptoms?

**Fig. 4:**
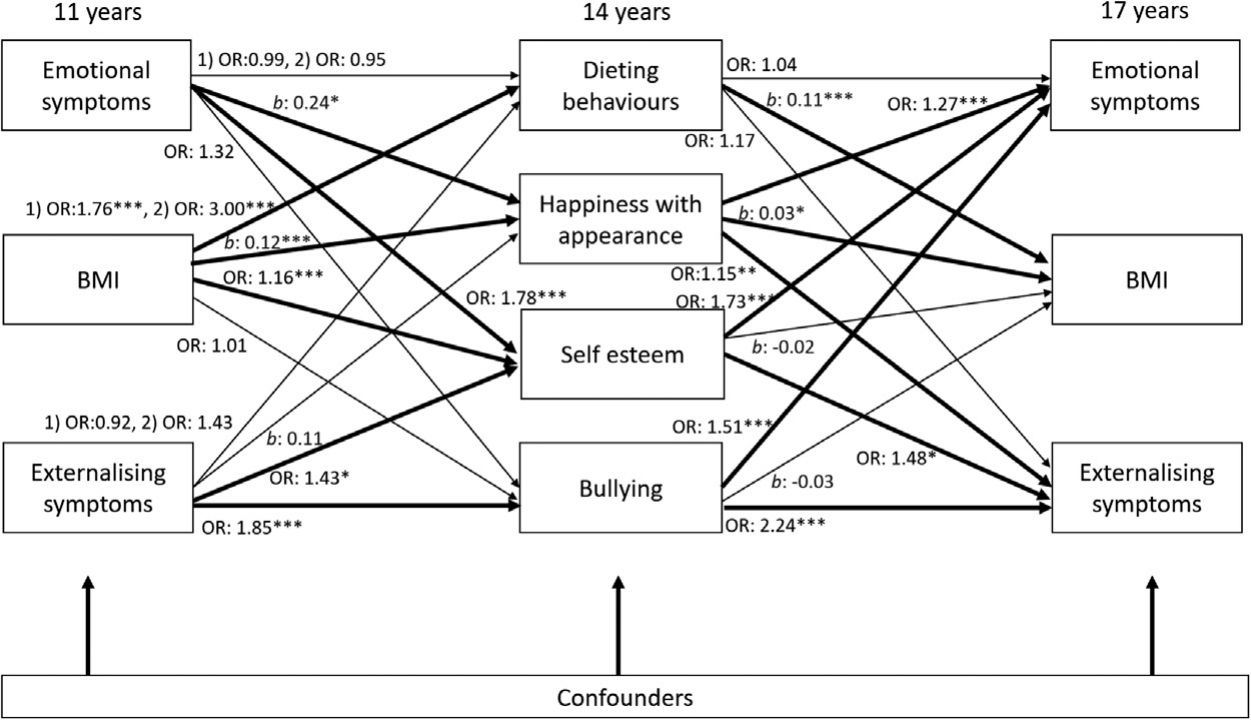
**Path model for mediation of cross-lagged associations from 11 years to 17 years between BMI Z-score and mental health symptoms for boys (N** = **6391)**. Notes: Odds ratios [OR] for logistic regression; Coefficients [*b*] for linear regression. All regressions are stratified by sex and adjust for cross–sectional associations between BMI Z-score, emotional and externalizing symptoms at baseline, and potential confounders: household income; maternal education; child ethnicity, early puberty and family structure). Regression estimates are weighted with sampling and attrition weights. ∗*p* < 0.05, ∗∗*p* < 0.01, ∗∗∗*p* < 0.001.

For boys, there was a significant role for happiness with appearance and self-esteem, but not dieting behaviours or bullying, in mediating the relationship between BMI and mental health symptoms (Fig. 4). Bullying was longitudinally associated with mental health symptoms, but not BMI. Dieting behaviours were longitudinally associated with BMI, but not mental health. At 11 years, BMI was associated with greater odds ratios for dieting behaviours (one behaviour OR 1.76, 95% C.I. 1.62 to 1.90; both behaviours OR 3.00, 95% C.I. 2.72 to 3.31). In turn, dieting at 14 years was associated with increased BMI z-score at 17 years (b 0.11, 95% C.I. 0.06 to 0.17). At 11 years, emotional symptoms (*b* 0.24, 95% C.I. 0.05 to 0.43) and BMI z-score (*b* 0.12, 95% C.I. 0.08 to 0.16) were related to greater scores of unhappiness with appearance at 14 years. In turn, happiness with appearance was associated with a greater odds ratio for emotional symptoms (OR 1.27, 95% C.I. 1.17 to 1.37), externalizing symptoms (OR 1.15, 95% C.I. 1.05 to 1.26) and higher BMI z-score (*b* 0.03, 95% C.I. 0.01 to 0.07) at 17 years. At 11 years, emotional symptoms (OR 1.78, 95% C.I. 1.30 to 2.44), BMI (OR 1.16, 95% C.I. 1.07 to 1.26), and externalizing symptoms (OR 1.43, 95% C.I. 1.04 to 1.96) were associated with greater odds ratios of low self-esteem at 14 years. In turn, self-esteem was associated with greater odds ratios for externalizing symptoms (OR 1.48, 95% C.I. 1.08 to 2.02) and emotional symptoms (OR 1.73, 95% C.I. 1.32 to 2.28) at 17 years. At 11 years, externalizing symptoms (OR 1.85, 95% C.I. 1.40 to 2.43) were associated with greater odds ratios of being bullied at 14 years. In turn, being bullied was associated with greater odds ratios for externalizing symptoms (OR 2.24, 95% C.I. 1.69 to 2.97) and emotional symptoms (OR 1.51, 95% C.I. 1.22 to 1.89) at 17 years.

**Fig. 5:**
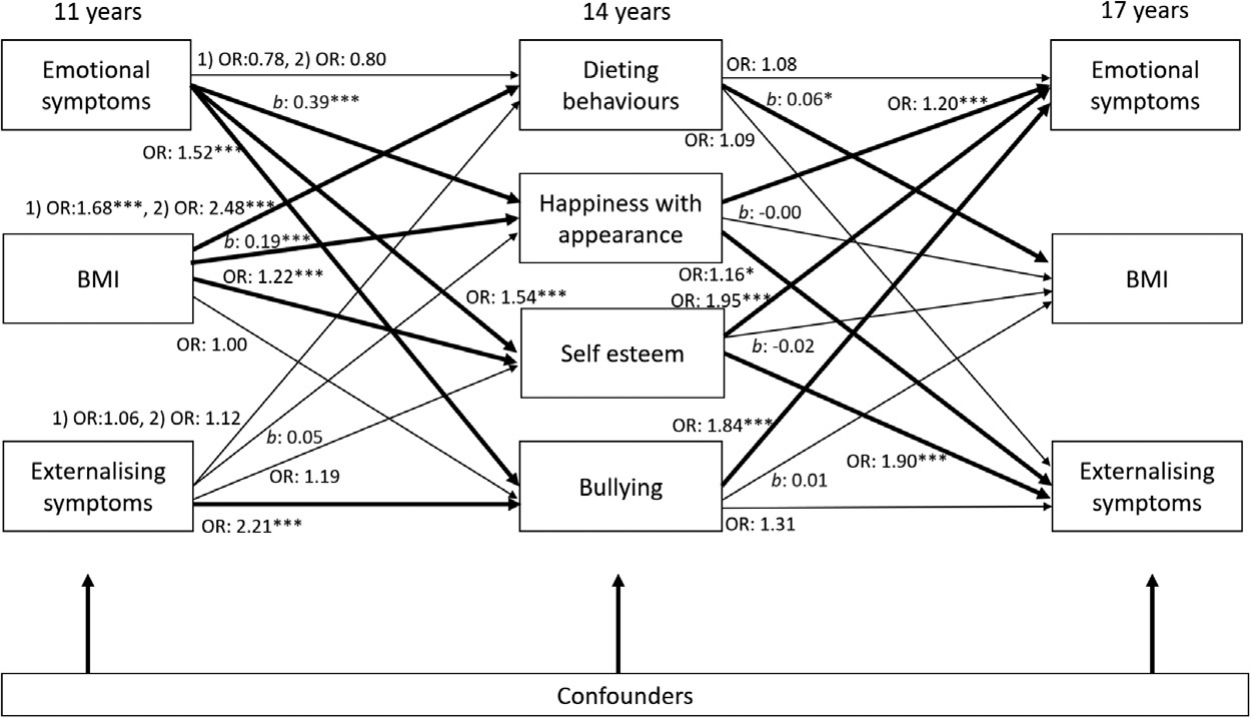
**Path model for mediation of cross-lagged associations from 11 years to 17 years between BMI Z-score and mental health symptoms for girls (N** = **6059)**. Notes: Odds ratios [OR] for logistic regression; Coefficients [*b*] for linear regression. All regressions are stratified by sex and adjust for cross–sectional associations between BMI Z-score, emotional and externalizing symptoms at baseline, and potential confounders: household income; maternal education; child ethnicity, early puberty and family structure). Regression estimates are weighted with sampling and attrition weights. ∗*p* < 0.05, ∗∗*p* < 0.01, ∗∗∗*p* < 0.001.

Similarly, for girls, there was a significant role for happiness with appearance and self-esteem, but not dieting or bullying, in mediating the relationship between BMI and mental health symptoms (Fig. 5). At 11 years, BMI was associated with greater odds ratios for dieting behaviours (one behaviour OR 1.68, 95% C.I. 1.53 to 1.83; both behaviours OR 2.48, 95% C.I. 2.30 to 2.68). In turn, dieting at 14 years was associated with increased BMI score at 17 years (b 0.06, 95% C.I. 0.01 to 0.11). At 11 years, emotional symptoms (*b* 0.39, 95% C.I. 0.22 to 0.57) and BMI (*b* 0.19 95% C.I. 0.14 to 0.23) were related to greater odds ratios being unhappy with appearance at 14 years. In turn, happiness with appearance was associated with greater odds ratios of emotional symptoms (OR 1.20 95% C.I. 1.13 to 1.28) and externalizing symptoms at 17 years (OR 1.16, 95% C.I. 1.03 to 1.29). At 11 years, emotional symptoms (OR 1.54, 95% C.I. 1.23 to 1.93) and BMI (OR 1.22, 95% C.I. 1.15 to 1.30) were related to greater odds ratios of low self-esteem at 14 years. In turn, self-esteem was associated with greater odds ratios for externalizing symptoms (OR 1.90, 95% C.I. 1.37 to 2.62) and emotional symptoms (OR 1.95, 95% C.I. 1.64 to 2.33) at 17 years. Emotional (OR 1.52, 95% C.I. 1.19 to 1.93) and externalizing symptoms (OR 2.21, 95% C.I. 1.53 to 3.19) at 11 years were associated with greater odds ratios of being bullied at 14 years. In turn, being bullied was associated with greater odds ratios of having emotional symptoms at 17 years (OR 1.84, 95% C.I. 1.51 to 2.23).

## Discussion

We hypothesised that for both, boys and girls, greater BMI at 11 years would be associated with later mental health difficulties by 17 years, and vice versa, via increases in dieting, happiness with appearance, low self-esteem, and frequent bullying. We found there was a significant role for happiness with appearance and self-esteem, but not dieting or bullying, in mediating the relationship between BMI and mental health symptoms. For boys and girls, high emotional symptoms compared to low/moderate symptoms and each increase in BMI z-score at 11 years were associated with greater odds of unhappiness with appearance and low self-esteem at 14 years. In turn, boys and girls who were unhappy with their appearance or had low self-esteem at 14 years were more likely to have emotional and externalizing symptoms at 17 years. Earlier unhappiness with appearance was also associated with higher BMI at 17 years for boys. For both boys and girls, dieting was longitudinally associated with BMI only and bullying was longitudinally associated with mental health symptoms only.

The direct effects in path models showed that, for girls, externalizing difficulties at 11 years were associated with higher BMI at 17 years. This adds longitudinal evidence to previous small, cross-sectional studies which have shown a higher prevalence of externalizing symptoms in adolescents with obesity.^36^

There was similar magnitude of associations for BMI with dieting, happiness with appearance and self-esteem for boys and girls. This contrasts with cross-sectional studies that suggest the impact of weight status on mental health is more profound for girls and young women than for their male counterparts.^25^ Dieting for both sexes, and unhappiness with appearance for boys, were also associated with later increases in BMI z-score, meaning that young people who had tried exercising or restricting food intake were more likely to have gained weight later in adolescence. This is consistent with the studies which have shown weight stigma increases the risk of obesity and that negative body image is harmful from the perspective of weight management as well as mental health.^17^

Emotional symptoms (anxiety and depression) were predictive of unhappiness with appearance and low self-esteem for boys and girls and bullying for girls. This is consistent with literature that has mainly focused on the impacts of depressive symptoms on body dissatisfaction and self-esteem.^14^^,^^37^ Yet, we also found boys with high externalizing symptoms (aggression and impulsivity) compared to low/moderate symptoms were more likely to report low self-esteem.

We found two studies that support bullying as a mediator of the relationship between BMI and later mental health.^38^^,^^39^ Van Vuuren and colleagues (2019) found that bullying was a significant mediator in the relationship between being overweight at 13 years and total socioemotional symptoms at 16 years (indirect effect OR: 2.3; 95% CI 1.5, 3.7) or suicidal thoughts (indirect effect OR: 2.1; 95% CI 1.4, 3.2). Our study is the first to simultaneously examine the opposite direction of association whilst analyzing temporal precedence. Although bullying was associated with previous and later emotional and externalizing symptoms, we found little evidence of it being a predecessor or successor of BMI.

To our knowledge, this study is among the first to use a nationally representative dataset to simultaneously test dieting, happiness with appearance, self-esteem, and bullying as potential pathways in the cross-lagged relationship between emotional and externalizing symptoms and BMI z-score across adolescence. Our study has significant strengths – firstly, we used data from a large, representative UK cohort making our findings generalisable to the wider population. Rich longitudinal data meant that we were able to simultaneously investigate three hypothesised mechanisms – dieting, happiness with appearance, self-esteem and bullying – which have been proposed as pathways in the cross-lagged relationship between mental health (emotional and externalizing symptoms) and BMI whilst adjusting for a wide range of potential confounding factors. To our knowledge, this is the first paper to have investigated multiple potential pathways across adolescence in this way. Using longitudinal path modelling, we were able to explicitly test hypothesised causal mechanisms, whilst controlling for cross–sectional associations between emotional and externalizing symptoms and BMI, adding weight to our findings.

However, as with all observational studies, attrition is a potential bias to consider. The MCS at baseline had a representative sample of 18,296 singleton children. Yet, by age 11 years 13,112 singletons were still productive in the cohort study. To minimise attrition bias, we used response weights to account for the loss of respondents up to age 11 years.^40^ The attrition weights adjust the sample composition to take account of the selective loss of respondents, for example, low-income families who may be less likely to remain in the cohort. More information on the MCS attrition weights can be found on the Centre for Longitudinal Studies website (https://cls.ucl.ac.uk/cls-studies/millennium-cohort-study/).

Generalised Structural Equation Modelling analyses the full, incomplete data via maximum likelihood estimation. This maximises the data included and lessens bias from missing data in listwise deletion. Of the 13,112 singletons still productive in the cohort at 11 years, 12,450 (95%) had some data on necessary variables and were included in the path analyses. Details of item missingness are given in Table A3, in the appendix. The majority of mediating variables had around 5% missing.

It is possible that the biases associated with parental reports mask stronger associations between weight status and mental health. Parent-reported mental health symptoms are commonly used in epidemiological studies^2^^,^^41^ and analysis of the SDQ has shown good cross-informant agreement and internal consistency,^28^^,^^42^ making it a useful tool in the absence of a clinical diagnosis. At both 11 and 17 years, the majority (95% at both waves) of parent reported respondents to the SDQ were the child's mother. We used the externalizing scale, combining the scores for conduct and hyperactivity/inattention problems. Goodman et al. (2010) advise that in low-risk, samples of the general population, individual subscales may not tap into the distinct aspects of child mental health. Avoiding the individual hyperactivity and conduct subscales and instead using the broader externalizing subscale is more appropriate when studying low-risk, non-clinical, epidemiological samples such as ours.^43^ In low-risk, samples of the general population you would expect scores for the Strengths and Difficulties Questionnaire be to positively skewed, with most young people scoring low and not having many difficulties. This is the distribution that we see for emotional and externalizing symptoms. We use a recommended cut point for higher symptoms indicating clinical significance (low/moderate 0–10 vs high symptoms 11–20).^31^

Children's BMI was objectively measured during home visits by trained interviewers at 11 and 17 years. While BMI is considered to be an acceptable but imperfect measure during childhood and adolescence, we were able to account for variation by sex and age using the validated and widely used sex and age adjusted Z-score.^32^

Self-reported bullying frequency may be subject to recall bias, and we were not able to take into account whether bullying was specifically due to young people's weight status or mental health symptoms. The frequency of bullying variable was positively skewed with most children experiencing infrequent bullying.

Tests for skewness and kurtosis showed that scores for the Rosenberg self-esteem scale at age 14 years was distinctly non-normal. This is consistent with previous reports of non-normal distribution of the Rosenberg self-esteem scale in the MCS.^44^ We are still limited by the data available as only five items from the Rosenberg self-esteem scale were administered in the MCS cohort, instead of the usual 10. We can see this from the questionnaire that was administered to young people.^45^ We were not able to take into account whether self-esteem was specifically due to adolescents’ feelings about perceived weight. However, dieting behaviours, happiness with appearance, and self-esteem scores were associated with weight status and each other in univariate analysis. Although we were able to capture aspects of internalized weight bias through self-reported dieting behaviours, happiness with appearance, and self-esteem, future research should adopt a more comprehensive approach to weight stigma assessment, as our findings are likely to be a conservative estimate of the true impact of weight stigma on the co-development of obesity and mental health, which has potential implications for clinical practice. As body image runs on a continuum, a scale of body satisfaction would have been the ideal for our study. However, a validated scale of body satisfaction was not administered in MCS. Instead, we take unhappiness with appearance and trying to lose weight/maintain weight to indicate potential negative body image. These items are markers of negative body image and the thin ideal which literature supports is the area of body dissatisfaction that predicts disordered eating and future weight status.^46^ However, we do acknowledge that body image concerns and related behaviours such as disordered eating often have complex presentations, especially in men.^47^

Adolescence is a critical window of development in which lifelong patterns of mental health and weight are established and can potentially be influenced.^1^ Early identification of high-risk groups is necessary for targeted interventions to be successful. We have shown that those exhibiting emotional or externalizing symptoms, who have tried dieting, or are unhappy with their appearance in adolescence are at greater risk of developing obesity than their peers and should therefore be prioritized in obesity prevention programmes. Adolescents with overweight or obesity and those unhappy with their appearance, exhibiting low self-esteem or are being bullied are at greater risk of mental health difficulties in adolescence and may benefit from early prevention strategies.

Given the powerful effect of happiness with appearance on mental health outcomes, it is imperative that young adolescents are supported to develop a positive body image, regardless of their weight.^48^ A holistic, early prevention strategy focussing on increasing positive body image is needed to encourage healthy physical and emotional development of children and adolescents. Young people understand from a very early age how society treats members of stigmatised groups, such as those deemed overweight or obese. This understanding of stigmatised others can impact young people's fear that they may be labelled and devalued and have harmful impacts on their physical and mental health.^49^^,^^50^ Weight stigma has been found to lead to discrimination in the workplace, education and healthcare settings.^17^^,^^51^^,^^52^ The narrative of “move more and eat better” stigmatises and oversimplifies the socially patterned, chronic relapsing condition of obesity and places the responsibility at the individual level.^53^^,^^54^ The latest Women and Equalities Committee enquiry into body image found that lockdowns during the Covid-19 pandemic worsened existing body image anxieties and increased risk of developing an eating disorder for many young people.^55^ Promisingly, body image education was introduced into the Relationships, Sex and Health Education (RSHE) curriculum in the UK in 2020.^56^ Over 100 medical and scientific organisations have already endorsed an international consensus statement published on World Obesity Day 2020 which pledges to eradicate weight stigma.^54^^,^^57^ Academic institutions, public health researchers and organisations, and the government should encourage positive body image and weight stigma education to facilitate a public narrative about obesity that is based on contemporary scientific evidence. Our findings reinforce calls for greater advertising and social media regulations to reduce weight stigma in adolescents.^58^

In conclusion, earlier mental health symptoms of aggression and impulsivity were associated with later BMI for girls and increases in BMI were associated with later symptoms of depression and anxiety for boys. We found evidence that the relationship between BMI and mental health was partially attributable to happiness with appearance and self-esteem.

## Contributors

HC and DH conceived the study idea. HC conducted the analysis and wrote the manuscript with support all authors. HC and DH verified the underlying data. DH and SS supervised the project.

## Data sharing statement

The data presented in this study are available from the corresponding author (HC) upon reasonable request for research purposes.

## Declaration of interests

SS reports grants from NIHR Applied Research Collaboration and The Daily Mile Foundation and is the president of the European Public Health Association. APS reports grants from Alicia Koplowitz Foundation and NHS England, payments for lectures and training from the Alicia Koplowitz Foundation and the University of Salamanca, and support for attending training in Child Mental Health for Spanish Trainees from the Alicia Koplowitz Foundation. DN reports grants from NIHR Applied Research Collaboration, Rosetrees Foundation, NIHR Biomedical Research Centre at Imperial College Healthcare NHS Trust, Healthcare Quality Improvement Partnership, Alicia Koplowitz Foundation and NHS England, payments from MindEd and the British Association of Psychopharmacology for lectures, royalties from Springer, and is a Clinical Advisor to BEAT.

## Appendix A Supplementary data

Supplementary Tables A1–A3
