# Supplementary material for: The role of dieting, happiness with appearance, self-esteem, and bullying in the relationship between mental health and body-mass index among UK adolescents: a longitudinal analysis of the Millennium Cohort Study
Source: eClinicalMedicine. 2023 May 22;60:101992. doi: 10.1016/j.eclinm.2023.101992 (PMC10314161; doi:10.1016/j.eclinm.2023.101992)
Supplement: Supplementary Tables A1–A3 [file mmc1.pdf]

Appendix

List of figures/tables:

1. **Table A1. Cross-lagged associations from 11 years to 17 years between BMI Z-score and mental health symptoms (N=12,508**
2. **Table A2. Path model for mediation of cross-lagged associations from 11 years to 17 years between BMI Z-score and mental health symptoms (N=12,450)**

Notes: [1] Logistic regression; [2] Linear regression [3] Ordinal logistic regression. All regressions are stratified by sex and adjust for cross-sectional associations between BMI Z-score, emotional and externalizing symptoms at baseline, and potential confounders: household income; maternal education; child ethnicity, early puberty, and family structure). Regression estimates are weighted with sample weights. Confidence intervals are in parentheses. *p<0.05, **p<0.01, ***p<0.001

3. **Table A3. Item missingness**

Table A1. Cross-lagged associations from 11 years to 17 years between BMI Z-score and mental health symptoms (N=12,508)

		Boys		Girls	
		**OR**	**(95% C.I.)**	**OR**	**(95% C.I.)**
BMI	→	Emotional[1]			
Z-score		1.09*	(1.01 to 1.18)	1.00	(0.93 to 1.07)
Externalizing	→	Emotional[1]			
Low/ Moderate		*Ref*			
High		1.26	(0.91 to 1.76)	1.29	(0.88 to 1.89)
BMI	→	Externalizing[1]			
Z-score		0.92	(0.82 to 1.03)	1.01	(0.90 to 1.13)
Emotional	→	Externalizing[1]			
Low/ Moderate		*Ref*			
High		0.94	(0.62 to 1.42)	0.91	(0.60 to 1.37)
		b	**(95% C.I.)**	***b***	**(95% C.I.)**
Emotional	→	BMI[2]			
Low/ Moderate		*Ref*			
High		0.09	(-0.05 to 0.23)	0.06	(-0.06 to 0.17)
Externalizing	→	BMI[2]			
Low/ Moderate		*Ref*			
High		0.17	(-0.01 to 0.34)	0.28**	(0.09 to 0.47)

Notes: [1] Logistic regression; [2] Linear regression. All regressions are stratified by sex and adjust for cross-sectional associations between BMI Z-score, emotional and externalizing symptoms at baseline, and potential confounders: household income; maternal education; child ethnicity, early puberty and family structure). Regression estimates are weighted with sample weights. Confidence intervals are in parentheses. *p<0.05, **p<0.01, ***p<0.001.

Table A2. Path model for mediation of cross-lagged associations from 11 years to 17 years between BMI Z-score and mental health symptoms (N=12,450)

**Indirect effects**	**Boys** **OR (95% C.I.)**		**Girls** **OR (95% C.I.)**	
Emotional symptoms →	Dieting behaviours[3]			
	One	Both	One	Both
Low/Moderate	*Ref*			
High	1.00 (0.74 to 1.35)	0.95 (0.67 to 1.33)	0.78 (0.55 to 1.12)	0.80 (0.59 to 1.08)
Emotional symptoms →	Self-esteem[1]			
High/Moderate	*Ref*			
Low	1.78*** (1.30 to 2.44)		1.54*** (1.23 to 1.93)	
Emotional symptoms →	Bullying[1]			
Low/ Moderate	*Ref*			
High	1.32 (0.98 to 1.77)		1.52*** (1.19 to 1.93)	
	***b* (95% C.I.)**		***b* (95% C.I.)**	
Emotional symptoms →	Happiness with appearance[2]			
Low/Moderate	*Ref*			
High	0.24* (0.05 to 0.43)		0.39*** (0.22 to 0.57)	
	**OR (95% C.I.)**		**OR (95% C.I.)**	
Externalizing symptoms →	Dieting behaviours[3]			
	One	Both	One	Both
Low/Moderate	*Ref*			
High	0.92 (0.64 to 1.31)	1.43 (0.9 to 2.14)	1.06 (0.59 to 1.96)	1.12 (0.69 to 1.81)
Externalizing symptoms →	Self-esteem[1]			
High/Moderate	*Ref*			
Low	1.43* (1.04 to 1.96)		1.19 (0.82 to 1.72)	
Externalizing symptoms →	Bullying[1]			
Low/ Moderate	*Ref*			
High	1.85*** (1.40 to 2.43)		2.21*** (1.53 to 3.19)	

Table A2. *continued*	**b (95% C.I.)**		**b (95% C.I.)**	
Externalizing symptoms →	Happiness with appearance[2]			
Low/Moderate	*Ref*			
High	0.11 (-0.08 to 0.30)		0.05 (-0.26 to 0.35)	
	**OR (95% C.I.)**		**OR (95% C.I.)**	
BMI →	Dieting behaviours[3]			
	One	Both	One	Both
Z-score	1.76*** (1.62 to 1.90)	3.00*** (2.72 to 3.31)	1.68*** (1.53 to 1.83)	2.48*** (2.30 to 2.68)
BMI →	Self-esteem[1]			
Z-score	1.16*** (1.07 to 1.26)		1.22*** (1.15 to 1.30)	
BMI →	Bullying[1]			
Z-score	1.01 (0.94 to 1.08)		1.00 (0.93 to 1.08)	
	**b (95% C.I.)**		**b (95% C.I.)**	
BMI →	Happiness with appearance[2]			
Z-score	0.12*** (0.08 to 0.16)		0.19*** (0.14 to 0.23)	
	**OR (95% C.I.)**		**OR (95% C.I.)**	
Dieting behaviours →	Emotional symptoms[1]			
None	*Ref*			
One/ Two	1.04 (0.92 to 1.18)		1.08 (0.97 to 1.19)	
Happiness with appearance →	Emotional symptoms[3]			
Score	1.27*** (1.17 to 1.37)		1.20*** (1.13 to 1.28)	
Self-esteem →	Emotional symptoms[1]			
High/moderate	*Ref*			
Low	1.73*** (1.32 to 2.28)		1.95*** (1.64 to 2.33)	
Bullying →	Emotional symptoms[1]			
Not bullied	*Ref*			
Bullied	1.51*** (1.22 to 1.89)		1.84*** (1.51 to 2.23)	
Dieting behaviours →	Externalizing symptoms[1]			
None	*Ref*			

Table A2. ***continued***

One/two	1.17 (1.00 to 1.37)		1.09 (0.89 to 1.33)	
Happiness with appearance ⟶	Externalizing symptoms[3]			
Score	1.15** (1.05 to 1.26)		1.16* (1.03 to 1.29)	
Self-esteem ⟶	Externalizing symptoms			
High/moderate	*Ref*			
Low	1.48* (1.08 to 2.02)		1.90*** (1.37 to 2.62)	
Bullying ⟶	Externalizing symptoms[1]			
Not bullied	*Ref*			
Bullied	2.24*** (1.69 to 2.97)		1.31 (0.95 to 1.81)	
	***b* (95% C.I.)**		***b* (95% C.I.)**	
Dieting behaviours ⟶	BMI Z-score [2]			
None	*Ref*			
One/two	0.11*** (0.06 to 0.17)		0.06* (0.01 to 0.11)	
Happiness with appearance ⟶	BMI Z-score [2]			
Score	0.03* (0.01 to 0.07)		-0.00 (-0.04 to 0.04)	
Self-esteem ⟶	BMI Z-score[2]			
High/moderate	*Ref*			
Low	-0.02 (-0.14 to 0.10)		-0.03 (-0.13 to 0.07)	
Bullying ⟶	BMI Z-score[2]			
Not bullied	*Ref*			
Bullied	-0.03 (-0.11 to 0.06)		0.01 (-0.08 to 0.10)	
**Direct effects**	**OR**	**(95% C.I.)**	**OR**	**(95% C.I.)**
BMI ⟶	Emotional[1]			
Z-score	1.00	(0.92 to 1.10)	0.91**	(0.84 to 0.98)
Externalizing ⟶	Emotional[1]			
Low/ Moderate	*Ref*			
High	1.23	(0.86 to 1.75)	1.02	(0.68 to 1.53)

Table A2. *continued*

BMI	⟶ Externalizing[1]			
Z-score	0.86**	(0.77 to 0.96)	0.92	(0.81 to 1.04)
Emotional	⟶ Externalizing[1]			
Low/ Moderate	*Ref*			
High	0.72	(0.47 to 1.12)	0.90	(0.59 to 1.38)
	b	**(95% C.I.)**	***b***	**(95% C.I.)**
Emotional	⟶ BMI[2]			
Low/ Moderate	*Ref*			
High	0.13	(-0.01 to 0.27)	0.03	(-0.09 to 0.16)
Externalizing	⟶ BMI[2]			
Low/ Moderate	*Ref*			
High	0.20*	(0.02 to 0.38)	0.29**	(0.10 to 0.48)

Notes: [1] Logistic regression; [2] Linear regression [3] Ordinal logistic regression. All regressions are stratified by sex and adjust for cross-sectional associations between BMI Z-score, emotional and externalizing symptoms at baseline, and potential confounders: household income; maternal education; child ethnicity, early puberty, and family structure). Regression estimates are weighted with sample weights. Confidence intervals are in parentheses. *p<0.05, **p<0.01, ***p<0.001.

Table A3. Item missingness

	% (n)
**Sex**	
Male	51.1 (6,634)
Female	48.9 (6,478)
Missing	0
Total	13,112
**Emotional symptoms at 11 years**	
Low/moderate	85.4 (11,223)
High	11.5 (1,405)
Missing	3.6 (484)
Total	13,112
**Emotional symptoms at 17 years**	
Low/moderate	60.8 (6,205)
High	29.5 (2,977)
Missing	9.7 (725)
Total	9,904
**Externalizing symptoms at 11 years**	
Low/moderate	89.2 (11,713)
High	8.3 (926)
Missing	3.5 (473)
Total	13,112
**Externalizing symptoms at 17 years**	
Low/moderate	82.4 (8,379)
High	7.9 (800)
Missing	9.7 (725)
Total	9,904
**BMI z-score at 11 years**	
BMI z-score	96.6 (12,696)
Missing	3.4 (416)
Total	13,112
**BMI z-score at 17 years**	
BMI z-score	79.9 (8,010)
Missing	20.1 (1,894)
Total	9,904
**Child ethnicity**	
White	84.3 (10,837)
Mixed	3.5 (378)
South Asian	7.1 (1,281)
Black	3.5 (423)
Missing	1.6 (193)
Total	13,112
**Early puberty**	
Not begun	83.0 (10,891)
Has begun	14.4 (1,832)
Missing	2.7 (389)

**Table A3.** ***continued***	
Total	13,112
**Household income**	
Top	20.1 (2,535)
Fourth	20.0 (2,767)
Third	20.0 (2,793)
Second	20.1 (2,519)
Bottom	20.0 (2,498)
Missing	0
Total	13,112
**Maternal education**	
Degree plus	10.1 (1,569)
Diploma	27.8 (3,959)
A levels	7.7 (1,085)
GCSE A-C	29.2 (3,605)
GCSE D-G	7.9 (893)
None	11.6 (1,376)
Missing	5.8 (625)
Total	13,112
**Age in months at 11 years**	
Age in months	13,112
Missing	0
Total	13,112
**Family structure**	
Two parents	73.4 (10,027)
One parent	26.6 (3,085)
Missing	0
Total	13,112
**Dieting behaviours**	
None	32.6 (3,625)
One	25.5 (2,754)
Both	36.7 (4,116)
Missing	5.1 (461)
Total	10,956
**Happiness with appearance**	
1 Completely happy	12.6 (1,417)
2	23.0 (2,605)
3	22.1 (2,428)
4	17.1 (1,888)
5	10.1 (1,099)
6	6.2 (658)
7 Not at all happy	3.5 (369)
Missing	5.6 (492)
Total	10,956
**Self-esteem**	
High/moderate	69.3 (7,788)

**Table A3.** ***continued***	
Low	25.2 (2,687)
Missing	5.5 (481)
Total	10,956
**Bullying**	
Not bullied	75.1 (8,391)
Bullied	19.7 (2,114)
Missing	5.2 (451)
Total	10,956
